# Introducing a specified on‐line multimodal prehabilitation approach for total knee replacement surgery candidates using data from the COVID‐19 pandemic: An exploratory field‐based, pre‐post, mixed methods implementation pilot study

**DOI:** 10.1111/jep.14186

**Published:** 2024-10-16

**Authors:** Laura Garland, Jamie Gibson, Rashida Pickford, Gareth D. Jones

**Affiliations:** ^1^ Physiotherapy Department Guy's and St Thomas’ NHS Foundation Trust Great Maze Pond London England UK; ^2^ Centre for Human and Applied Physiological Sciences (CHAPS), King's College London Guy's Campus London UK

**Keywords:** exercise, implementation, knee surgery rehabilitation, preoperative care, telerehabilitation, treatment efficacy

## Abstract

**Rationale:**

Individuals waiting for total‐knee‐replacement surgery are at risk of developing morbidities and frailty which may affect their postoperative recovery. Multi‐modal prehabilitation could mitigate these unintentional effects.

**Aims and Objectives:**

To implement and evaluate a specified online multi‐modal prehabilitation intervention in patients waiting for total‐knee‐replacement surgery in a large urban acute hospital trust.

**Method:**

A non‐randomised, pre/post analysis implementation pilot with a nested qualitative study was conducted and is reported following the standards for implementation studies (StaRI) guidance. Of 35 listed cases, 12 (34%) were eligible, recruited, and completed an 8‐week multi‐modal online intervention incorporating 5 modalities (i) cardiovascular exercise, (ii) strength/balance function, (iii) smoking cessation, (iv) opioid use, (v) nutritional intake. Interventions were specified using the Rehabilitation Treatment Specification System, where rehabilitation treatment theory accounts for discrete treatment components. Two participated in an online qualitative interview post‐intervention. Process evaluation included intervention fidelity, eligibility/recruitment/retention rates, and clinical outcomes included knee function, frailty, gait velocity, anxiety/depression, and quality of life.

**Results:**

Five participants (42%) completed the intervention and were retained at follow‐up. The intervention was delivered online at specified doses, frequency/durations indicative of high respective adherence, quantity, and exposure fidelity.

There was significant improvement in median oxford knee score (*p* = 0.015), gait velocity (*p* = 0.040) and anxiety (*p* = 0.023). The interview revealed 5 themes; surgery preconceptions, motivation, acceptability, postoperative experiences, and future recommendations confirming acceptance of the intervention by virtue of adhering to the treatment exposure delivered as planned.

**Conclusion:**

The specified multi‐modal prehabilitation was acceptable, implementable, and demonstrated evidence of preliminary efficacy. Further experimental pilot work that represents the spectrum of frailty, obesity, quality of life, and comorbidities associated with total‐knee‐replacement surgery is indicated.

## 1 INTRODUCTION

Joint replacement surgery is cost‐effective for end‐stage degenerative joint disease.^1^ In the UK there were >100,000 primary unicompartmental or total knee replacement (TKR) procedures annually between 2015 and 2019 with osteoarthritis (OA) implicated in 97% of cases.^2^, ^3^ COVID‐19 pandemic‐related surgery suspensions meant annual surgery rates halved in 2020^2^ leading to TKR patients facing waits of ≥12 months.^4^

Age, OA incidence, and disability symptoms^5^, ^6^ correlate with frailty, a syndrome associated with vulnerability and depression.^7^ Frailty affects 9% of people aged >65 years and 25–50% >85 years.^8^ Preoperative frailty is associated with increased opioid use^9^, ^10^ leading to opioid dependence, hyperalgesia, and suboptimal physical function post‐surgery.^11^, ^12^ Government advice during the pandemic urged vulnerable individuals to avoid social contact and paradoxically influenced their decisions on, and access to, healthcare needs.^13^ Social isolation policies globally reduced physical activity, increasing the risk of frailty.^14^ Given that ~20% of arthroplasty patients were socially isolated pre‐COVID‐19,^15^ and isolation is linked with worsening health in older people,^16^ enforced COVID‐19 isolation increased risks of frailty and associated morbidities. One approach to mitigate these unintentional effects is to provide prehabilitation following the decision to list for TKR.

The purpose of prehabilitation is to optimise patients’ capacity before a planned intervention to withstand the incoming stressor and prevent adverse events post‐intervention.^17^ Although prehabilitation is part of enhanced recovery after TKR surgery (ERAS) approaches, it is often limited to educative interventions.^18^ A more thorough approach is multimodal prehabilitation^19^ which includes not only exercise/education, but also ingredients designed to address poor nutrition, dysfunctional habits (e.g., smoking), and cognitions (e.g., anxiety).^20^ Prehabilitation is endorsed by health professionals who prefer to conceive of a preparation list rather than a waiting list.^21^

Recent meta‐analyses have shown ERAS approaches in major joint surgery are safe and efficacious including reducing hospital length of stay (LOS) and complications.^22^ While this sentiment is echoed in UK national guidance based on experimental trials, they included supervised prehabilitative exercises/advice without other recommended modes (e.g., smoking cessation or optimal nutrition).^23^ This suggests that prehabilitation could be both more efficacious and better specified.

In fact, prehabilitation, like most fields of rehabilitation, remains poorly specified where interventions are conceived as a black‐box.^24^ The Rehabilitation Treatment Specification System (RTSS) offers a theoretical framework to address the problem. It enables rehabilitation clinicians to specify treatment based on treatment theories where active treatment ingredients hypothesised to directly change a specified treatment target via a mechanism of action are specified.^25^ While reports of specified prehabilitation in spinal surgery exist,^26^ specified TKR multi‐modal prehabilitation remains elusive.

In addition to efforts to specify rehabilitation interventions including prehabilitation, another consideration is the environment in which prehabilitation is delivered. Telerehabilitation or delivering interventions online have gained attractiveness and an acceleration in implementation since the COVID‐19 pandemic.^27^, ^28^ Compared to the traditional in‐person environment, online consultations are particularly attractive to clinicians and funders because of the appeal of time, space, and cost savings.^29^, ^30^ Online interventions are also attractive as countermeasures to what patients express as barriers to participation during in‐person rehabilitative interventions, for example travel time, transport requirements, cost, and their interference with other employment, caring, or social commitments.^31^, ^32^

**Figure 1:**
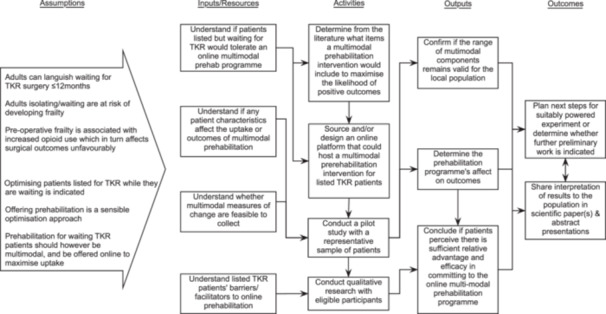
Logic model representing the pilot project programme theory.

Individuals waiting for TKR surgery are at risk of developing morbidities and frailty which may affect their postoperative recovery. Implementing and assessing the efficacy of a specified multi‐modal prehabilitation robust enough to be delivered despite government isolation policies is therefore required. The aim of this study was to evaluate implementing a specified online multi‐modal prehabilitation intervention programme for TKR patients following guidance on developing complex interventions,^33^ and simultaneously provide preliminary evidence on its clinical efficacy. The programme theory, using a logic model,^34^ claims that specified multi‐modal prehabilitation implemented in the local context for socially isolated listed patients waiting for TKR surgery will sufficiently target and therefore maintain/improve physical functions, behaviors, and cognitions which otherwise would experience a gradual loss of in‐built reserves resulting in frailty. The mechanisms underlying the claim are based on the effects of targeting cardio‐respiratory reserve capacity which increases whole‐body tolerance to systematic perturbations major surgeries cause,^35^ while accepting that exercise training alone is insufficient to attenuate the stress response when nonoptimal nutrition, coping behaviors, and pre‐morbid lifestyle choices are taken into account (Figure 1). The objectives were to:

Determine if an online multimodal programme could be delivered at a specified dose for TKR patients listed for surgery and whether patients would accept the intervention by virtue of adhering to the treatment exposure delivered as planned.

Understand participants’ barriers and facilitators to conducting the programme online.

Determine if there is any statistically significant change in multimodal clinical measures between the duration of the planned intervention and at follow‐up.

## 2 METHODS

### 2.1 Design and ethics

This was a non‐randomised, pre/post analysis pilot with a nested qualitative study conducted in a large urban public NHS trust. The local governance committee provided ethical approval (project no: 11272). All patients provided consent to participate. Reporting is in accordance with the standards for implementation studies (StaRI) guidance.^36^, ^37^

### 2.2 Participants

Patients (≥18 years) prospectively listed for primary unilateral elective TKR between September 2020 and April 2021 were eligible. Exclusions were possible delirium (4AT>3^38^), no access to online electronic devices, knee revisions, or other previous ipsilateral leg surgery within 12‐months.

### 2.3 Intervention

An 8‐week online multimodal prehabilitation intervention was delivered by a trained physical therapist (PT). It was designed to modulate activity and life‐style behaviors based on national guidance^39^ and UK consensus^40^ across 5 modalities (i) cardiovascular exercise, (ii) strength and balance function, (iii) smoking cessation, (iv) opioid use, and (v) nutritional intake. The intervention was specified using the RTSS.^25^

### 2.4 Cardiovascular exercise, strength & balance function

The online exercise intervention followed the PEAK program.^41^ It consists of 5 one‐to‐one consultations delivered via video‐conferencing in addition to a directed, volitional, home exercise programme (8‐weeks, 3 sessions/week, Supplementary Table 1).

### 2.5 Smoking cessation

Smoking cessation support was offered based on national guidance^42^ using the 3 A's protocol^43^ which incorporates *asking* whether an individual smokes, *advising* on the effects and risks associated with smoking, and *acting* upon the response. Patients were also referred to ‘opt‐out’ services and provided with locally assured written material (Supplementary Table 2).

### 2.6 Opioid use and pain management

Advice about the detrimental risks associated with high opioid use was offered in parallel with pain management advice. High dose users were encouraged to arrange a primary care physician appointment for analgesia review if they have not already done so within 3 months (Supplementary Table 3).

### 2.7 Nutritional intake

For participants living with overweight (BMI 25.0–30.0 kg.m^−2^) or obesity (BMI>30.0 kg.m^−2^), written lifestyle weight management advice was offered to participants.^44^ For under nourished participants (BMI<18.5 kg.m^−2^), dietary information advice was offered^45^ (Supplementary Table 4).

### 2.8 Study flow

**Figure 2:**
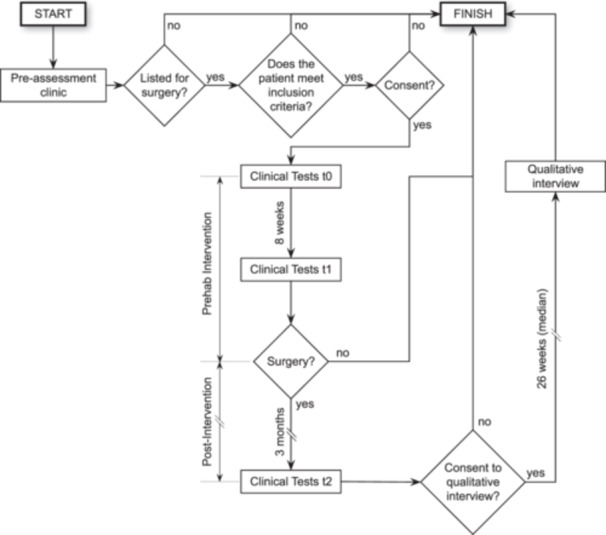
Study flow, Patients screened for participation at their pre‐assessment clinic where baseline (t0) measures taken and prehabilitation intervention offered if they fulfilled the inclusion criteria. Clinical tests were repeated at ≥8 weeks (t1) and the intervention continued until their signature surgery was completed. Finally, clinical tests were repeated 3 months post‐surgery (t2), whereafter patients were invited to participate in a qualitative interview.

Baseline clinical measures were made in‐person if the decision to list for surgery was made (t0) and were repeated 8‐weeks later (t1), and 3‐months post‐surgery (t2). All participants were invited to take part in a focus group after recovering from their surgery (Figure 2).

### 2.9 Process evaluation

A summative process evaluation was utilised to judge whether the intervention was implemented as planned, to determine the extent to which participants were exposed,^46^ and the preliminary intervention efficacy with respect to programme theory and context according to a recognised framework (CFIR)^47^ (Supplementary Table 5) using quantitative and qualitative data outlined below.

### 2.10 Quantitative data

Implementation data included rates of eligibility (% eligible from TKR surgery list), recruitment (% eligible recruited), and retention (% recruited completing intervention). Intervention fidelity was assessed in the adherence, quantity, and exposure domains (Table 1).^48^

**Table 1:** Summary of prehabilitation treatment fidelity assessments according to Sanetti et al 2021.^48^

Fidelity Term	Definition	Assessment
Adherence	The extent to which the intervention steps were implemented at the specified dose	% of dosing parameters delivered to recruited patients when they attended
Quantity	The frequency/duration with which the intervention was delivered	% of planned online and in‐person patient encounters
Exposure	The frequency/duration recipients to which participants received the intervention	% patients completing/participating in the intervention online/in‐person

Intervention efficacy data were collected at t0, t1, and t2 and included self‐reported knee pain and function using the Oxford Knee Score (OKS)^49^ a 12‐item numerical scale (0–4 (best)) rating physical function and pain in the preceding 4 weeks (max score 48). Frailty was determined if standardized^50^ dominant hand grip strength was ≤lower 95% CI of age‐norm.^51^ Knee range, strength, and standing balance were measured by knee flexion/extension in sitting (long‐arm goniometer; Lafayette, IN, USA), 1‐min sit‐to‐stand test,^52^ and the clinical test of sensory interaction and balance (CTSIB)^53^ respectively. Gait velocity as a surrogate of ambulatory function, was assessed using a 4 m self‐selected timed walk.^54^

Cognition was screened using the 4AT^38^ where scores 1–3 suggest nonspecific cognitive dysfunction. Anxiety and depression were assessed using the hospital anxiety and depression (HAD) scale^55^ where ≥8/21 reveals at least mild burden for both subscales,^56^ and QOL using the EQ‐5D‐5L questionnaire.^57^ Analgesia was assessed using an ordinal scale of prescribed opioids (low (<30 mg)), medium (31‐60 mg), high (61–120 mg), and very high (>120 mg).^58^ If participants were ex‐smokers, their number of quit‐days were recorded. Hospital LOS was days between TKR surgery‐hospital discharge. Finally, ptostoperative adverse events were assessed by count of patient incident reports filed between 10 October 2020 and 28 November 2021.

### 2.11 Qualitative data

Participants completing the prehabilitation programme were invited to a 65‐min online interview in April 2022, facilitated by an independent PT. Those who accepted represented a subgroup who volunteered to reveal their experiences of the intervention. The lead author completed field‐notes. A topic guide (Supplementary Table 6) explored recollective perspectives, feelings, attitudes, and experiences. The interview was recorded with participant consent and transcribed verbatim by the lead author.

The PTs are both experienced (>10 years experience) musculoskeletal professionals. They perceive prehabilitation in TKR as important. Participants were informed of their professional roles during recruitment and the interview. Both PTs kept a reflexive account after the focus group to assess how prior knowledge, attitudes, and perception influenced data analyses.

### 2.12 Data analysis

Friedman tests^59^ were used to assess the difference in all clinical tests within participants across t0, t1, and t2 except analgesic use and smoking where a related‐sample Cochran's test was used.^60^ If significant differences were revealed, pairwise comparisons were performed with a Bonferroni correction. For all statistical tests (SPSS v24, IBM Corp, Armonk, NY, USA) significance was assumed at ≤0.05.

Interview transcripts were assessed independently using an inductive thematic analysis approach.^61^ Main themes and associated sub‐themes were color‐coded to synthesise the emerging themes using proprietary software (Microsoft Excel, Redmond, WA, USA). After duplicates were removed, consensus was reached on a final set of themes. The trustworthiness of data was ensured by inviting participants to review the coding themes and summaries affirming their thoughts were represented correctly.^62^

## 3 RESULTS

### 3.1 Recruitment

There were 35 patients listed for TKR surgery over the 7‐month recruitment period. Twelve were eligible (34% eligibility rate) and were recruited after being offered prehabilitation (100% recruitment rate) (mean ± SD age 64 ± 14 years, 4(33%) male, 8(67%) female). Ineligible patients consisted of those either not living locally (*n* = 7), speaking English (*n* = 1), having access to online devices (*n* = 2), being medically fit (*n* = 1), or those receiving knee revision surgery (*n* = 4), or choosing not to participate (*n* = 3). Primary 2020/2021 UK TKR data (all type) reveals consistent mean ± SD age (years) and sex of 70 ± 9 (57% female) and 70 ± 9 (56% female) respectively meaning our sample was representative of age/sex.^63^, ^64^ Most identified as White British, were university educated, nonsmoking, retired, not frail nor obese, had low comorbidity, and while required social support were all community ambulators (Table 2).

**Table 2:** Recruited participant characteristics (*n* = 12).

	Characteristic	Data
		12
	Age (yrs)	64 (±14)
	Height (m)	1.66 (±0.08)
	Weight (kg)	81.50 (±12.34)
	BMI (kg.m^−2^)	29.67 (±3.65)
	Lean body mass (kg)^a^	52.30 (±9.34)
	Hospital LOS (days)	2.41 (±1.66)
Sex	Female	8 (67%)
	Male	4 (33%)
Ethnicity	South Asian	1 (8%)
	Black African	1 (8%)
	White British	7 (58%)
	White Other	3 (25%)
Education^b^	GCSE/O Levels	4 (33%)
	Diploma	1 (8%)
	University Degree	7 (59%)
Employment	Employed	3 (25%)
	Retired	6 (50%)
	Student	1 (8%)
	Volunteer	1 (8%)
	Unemployed	1 (8%)
Social Support	Yes	11 (92%)
	No	1 (8%)
Planned Replacement Surgery	Total Knee	10 (83%)
	Unicompartmental Knee	2 (17%)
Community Ambulation	Independent	12 (100%)
Smoker	Yes	1 (8%)
	No	9 (75%)
	Previously	2 (17%)
Comorbidity	Median (IQR) Charlson^c^	3 (2‐3)
Analgesia (dose)^d^	No	2 (17%)
	Low	6 (50%)
	Medium	3 (25%)
	High	1 (8%)

All data are mean (±SD) or counts (%) unless otherwise stated.

Abbreviations: BMI, body mass index (proportion of the squared mass with respect to height); GCSE, general certificate of secondary education equivalent to high‐school education; LOS, length of hospital stay; O Levels, precursor to GSCEs equivalent to high school education.

^a^ Estimated lean body mass.^65^

^b^ Highest education attainment noted.

^c^ Charlson comorbidity index.^66^

^d^ Classification Opioid subgroups (low (<30 mg), medium (31–60 mg), high (61–120 mg), and very high (>120 mg).^58^

Five patients completed the 8‐week intervention and 3 month postsurgical follow up (42% retention rate) (Table 3 below). Median intervention time (range) was 9 (8–13) weeks. Reasons for drop out were; did not proceed with surgery (*n* = 1), other illness requiring treatment (*n* = 2), and surgery offered before completing prehabilitation (*n* = 4). Only two patients participated in the focus group (40%; 2 declined and 1 re‐located) which was an insufficient number to engender group interactions, it was therefore conceived as a group interview.

**Table 3:** Characteristics of participants (*n* = 5) completing intervention and change in measures of outcome.

Measure		t0	t1	t2	sig
Mean (± SD) age (yrs)		60 (±22)	–	–	–
Time to surgery^a^ (days)		84 (12–204)	–	–	–
Prehabilitation period (days)		57 (55–62)	–	–	–
Hospital LOS (days)		3 (2–4)	–	–	–
4AT		0 (0–0)	–	–	–
Planned Surgery	Total	4 (80%)	–	–	–
	Uni	1 (20%)	–	–	–
Height (m)		1.69 (1.58–1.78)	–	–	–
Weight (kg)		81.0 (72.4–95.0)	79.0 (73.0–95.0)	75.0 (73.0–95.0)	ns
BMI (kg.m^‐2^)		29.6 (29.1–32.4)	29.6 (29.2–31.6)	29.6 (29.2–30.0)	ns
Lean body mass (kg)^b^		47.7 (46.8–67.3)	47.8 (46.3–67.3)	47.8 (45.3–67.0)	ns
Smoker (Yes)		1 (20%)	0 (0%)	0 (0%)	ns
Number of days quit smoking		0 (0–0)	0 (0–0)	0 (0–0)	ns
Oxford Knee Score^c^		21 (19–24)	25 (24–29)	36 (35–38)	[*]
Grip Strength (kg)		35 (30–40)	36 (32–40)	38 (37–38)	ns
Frailty (Yes)		1 (20%)	1 (20%)	0 (0%)	ns
Gait Velocity (m.s^‐1^)		0.98 (0.95–1.05)	1.48 (1.03–1.54)	1.25 (1.05–1.29)	[*]
CTSIB Balance (s)^d^		100 (96–100)	99 (99–110)	111 (108–112)	ns
60 s STS (count)		21 (16–24)	24 (21–25)	21 (20–23)	ns
EQ. 5D5L		0.30 (0.21–0.60)	0.40 (0.28–0.70)	0.17 (0.05–0.20)	ns
EQVAS		70 (55–85)	80 (75–85)	80 (80–90)	ns
HADS (Depression)		4 (1–8)	9 (4–9)	8 (3–8)	ns
HADS (Anxiety)		5 (3–7)	5 (3–7)	4 (2–6)	[*]
Analgesia^e^	No	3 (60%)	3 (60%)	2 (40%)	ns
	Low	2 (40%)	2 (40%)	3 (60%)	

All data are median (IQR) or counts (%) unless otherwise stated.

Abbreviations: BMI, body mass index; HADS, hospital anxiety and depression scale; LOS, length of stay; ns, not significant; Sig, statistical significance; STS, sit‐to‐stand.

^a^ Time from listing to surgery.

^b^ Lean body mass calculated using an online calculator with the Boer Formula.^65^

^c^ Pain & function subscale (max score 48).

^d^ Clinical test of sensory integration in balance.

^e^ Analgesia opioid use based on ordinal criteria.^58^

* Indicates statistical difference across the 3 time points at the ≤0.05 level; 4AT – The 4 ‘A's rapid test of cognition.^38^

### 3.2 Fidelity

RTSS dosing parameters were either delivered as specified or were not applicable in modes specified as representation treatment groups (Supplementary Table 1–4), meaning there was 100% adherence fidelity in these cases. In contrast, dosing parameters for organ function/skills and habit treatment groups were partially tailored to patients, and adherence fidelity is therefore less clear but was estimated at between 80% and 100% which is considered high.^67^ Quantity and exposure fidelity were also high with intervention encounters delivered for 100% of the planned frequency/duration, and 100% of patients completing/participating in the intervention online/in‐person. Therefore, the implementation process was carried out as planned in line with our logic model.

### 3.3 Multimodal prehabilitation intervention outcomes

There were significant differences across t0, t1 and t2 in median Oxford Knee Score [*χ*2(2) = 8.40; *p* = 0.015] with pairwise comparisons revealing a significant difference between t0 and t2 (*p* = 0.013). In addition, while there were significant differences across t0, t1, and t2 in median (IQR) gait velocity [*χ*2(2) = 6.42; *p* = 0.040] and in the HADS anxiety subscale [*χ*2(2) = 7.54; *p* = 0.023], post‐hoc testing failed to reveal any significant differences between any combinations of time points in either variable. There were no other statistical differences across t0, t1 and t2 in any other variable (Table 3) including smoking behaviour despite the one smoker reporting 0, 36, and 137 quit‐days at t0, t1, and t2 respectively.

### 3.4 Adverse clinical events

No adverse events were reported.

### 3.5 Qualitative results

A total of 59 codes were generated from interview. Codes were grouped into five themes which included preconceptions towards surgery, motivation, acceptability, postoperative experiences, and future recommendations (Table 4).

**Table 4:** Qualitative themes: Each theme is numbered and described with example quotes provided.

No	Theme	Description	Example Quotes
1	Experiences and preconceptions of rehabilitation and surgery	Participants reported negative experiences pre‐operatively to poor communication across different orthopedic specialties, and recalled not being listed for TKR surgery due to not taking any analgesia. Further to this, pre‐operatively the participants expressed concerns over being prescribed physiotherapy exercises for some time until their knee had sufficiently deteriorated and wondered if earlier surgical intervention would omit degeneration on other joints, reducing the risk of further joint surgery.	“when I first saw the surgeon about my knee surgery and he wouldn't put me on the waiting list at first because I didn't take painkillers and I think that's one thing you should not be promoted madly because you shouldn't be on painkillers long term.” (Participant 1) “I was asked to do physio exercises until it got to a state where you're so bad. It would have been better to have had the operation earlier, and then perhaps I wouldn't have had to have all the other operations. (Participant 1)
2	Motivation and compliance	Participants were motivated to adhere to the exercises and capable of doing them, but one participant reported their drive to change behavior waned after a while with feeling they could have done more. They also spoke of wanting to lose weight before the TKR surgery but reflected on low self‐efficacy with this. In contrast, the other participant reported to be highly motivated and competitive with themselves with regard to the exercises. There was a discrepancy between participants and their thoughts on expected exercise dose and dose of exercise prescribed.	“I was not as good as I should have been and didn't try and do more than I could” (Participant 2) “I planned to lose weight, but I didn't manage very well, as I'm really rubbish at that.” (Participant 2) “I was doing them diligently and was competitive with myself” (Participant 1) “I think you were expecting too many of each exercise” (Participant 2)
3	Acceptability of the Multimodal Intervention	Each participant felt happy to be involved in the programme and thought it was beneficial and improved their knowledge and understanding on OA, exercise and lifestyle changes. Although, neither participant changed their diet, one participant reported as already having dietary habits that were successful in achieving weight loss. Both participants said they would recommend the programme to other patients waiting for TKR surgery.	“I was very happy to be involved with it, a good idea and knew it would be beneficial” (Participant 2) “I didn't really change my diet very much, from the beginning I've been brainwashed at a young age. What I can and can't have, when I can have a drink and that, so it didn't really change a lot for me” (Participant 1) “I learnt a lot, would recommend it and felt I got more information online compared to a normal appointment” (Participant 2)
4	Postoperative Experience and Care	Postoperatively both participants reported positive experiences with the PTs on the ward, however, aftercare with regard to communication with the GP about analgesia, dressing changes and staple removal to be negative. One participant felt they lacked being informed as to when to take their analgesia on discharge home, resulting in them being in a lot of pain. Both participants agreed that exercises prescribed pre‐ and postoperation to be an essential part of recovery and recommended support at home on discharge. Both participant's reported recovery and returning to work took longer than expected.	“On the ward the physiotherapists were very good, but it did not dawn on me until I got home that I had to continue to take medication to help with the pain. I was in so much pain overnight. I tried to speak to my doctor but they didn't help. If it wasn't for my pharmacy I don't know what I would have done.” (Participant 2) “I thought the operation and Physio's were very good. I had my staples taken out at the GP. I asked if they could put the cold stuff on to freeze it but they said I had to buy it myself, well had I known I would have bought it. It was so painful” (Participant 1) “I cannot stress enough due to doing the exercises pre and post to help straighten and bend the knee which was key for recovery.” (Participant 2) “I was going to be back at work within six weeks but in fact I was off for four months” (Participant 1)
5	Recommendations for future iterations	Both participants felt that the exercises should be more bespoke and given a choice so they can choose their exercises, which are tailored towards their current fitness levels and needs. The programme should also include further information about education on the importance of adherence to exercise preoperatively and your recovery will be quicker post‐surgery. They also recommended promoting a balanced diet and include education about expectations of postoperative pain, including advice on pain control, that support at home is required and what to expect on discharge home, as well as timeframes of recovery. Both participants claimed they managed well with the online programme but would prefer the option for in‐person due to a perceived superiority in exercise supervision and responsiveness to questions, so it would be good to offer both online and in‐person options for the programme, in case people did not wish to attend the hospital.	“Exercises should be personalised & more information about our previous experience should be collected so exercises can be more tailored. Actually, you should provide a menu of exercises so patients can choose which ones they do, while listening to music, that motivates me.” (Participant 2) “I think you need to make people aware our important it is to do these exercises before the operation and include some education about if you adhere to this programme your recovery will be quicker.” (Participant 1) “I was eating healthy before but promoting a balance diet and give advice about pain after the surgery would be good. I can't emphasise enough that you need someone when you go home and include what to expect when going home. I thought I was going to be back at work in six weeks but it was about 4 months in the end so advice on how long to get better would be good too.” (Participant 2) “I think it's been very helpful being online, especially over COVID, but I think it seems better in person, as it's a better way to gauge if you are doing it right and you can ask questions more easily.” (Participant 1)

## 4 DISCUSSION

### 4.1 Main findings

The online multimodal prehabilitation was acceptable, implemented as planned, and demonstrated preliminary clinical efficacy in reducing knee pain and function, gait velocity, and anxiety. This study is novel because prehabilitation was specified using the RTSS meaning it can be communicated among rehabilitation professionals using a common language and therefore be repeated/modified and tests of efficacy/effectiveness can be designed.^25^ It also means mechanisms of action can be developed which is pertinent in OA given the mechanisms modulating pain and function via exercise therapies remain unclear.^68^

### 4.2 Fidelity and qualitative insight

While our sample was age/sex representative for TKR patients, it was not for frailty, comorbidity, BMI, or QOL presumably because lower‐risk patients were prioritised for surgery. This means the programme theory needs to be prioritised for diversity of the population in the “outer setting” CFIR^47^ context. We did not include programme implementation observation (either directly or recorded), which is considered gold standard for assessing treatment quality fidelity.^48^ Irrespectively, we were able to confirm the PT adhered to implementing prehabilitation steps at specified therapeutic doses, and delivered the correct quantity (frequency/duration) online, to which patients were adequately exposed.

The qualitative data partially supported our fidelity findings. Patients expressed initial fear yet still opted for prehabilitation. They reported increased knowledge in osteoarthritis management and willingness to recommend the programme. However, concerns about the intensity/frequency of the programme were conveyed, indicating patient exposure issues. Customizing the programme may improve exposure and assessing fidelity quality, including self‐directed exercises, could inform adjustments to the programme such as emphasising cognitive processing treatment components.

While patients expressed acceptance of the online intervention and favoured attaining educative information online compared to in‐person, they also recommended utilising in‐person consultations for other elements of the intervention. They recognised the attributes of online delivery especially during COVID‐19 isolation but thought in‐person consultations benefit timely provision of feedback to their questions and their performance, raising the possibility they expressed a preference towards blended online/in‐person intervention designs. These qualitative insights frame the dangers in being persuaded to move rapidly to online‐only interventions because of the interaction between compelling cost benefits to organisations on the one hand, and cost benefits to patients on the other. The development of interventions by clinicians, researchers, and funders should consider the socioeconomic factors attributed to the patients they serve, such as lack of financial agency to purchase necessary hardware or self‐monitoring equipment, because not doing so could affect the digital divide and existing health inequalities where underserved groups in society are less likely to access telehealth.^69^

It is also yet to be determined whether online rehabilitation is superior to in‐person interventions. For example, a recent literature review and metanalysis of superiority trial studies of post operative TKR patients that compared the effect on functional outcomes between telerehabilitation and conventional in‐person rehabilitation revealed equivocal results.^70^ While the review did reveal two studies providing compelling cost benefit for telerehabilitation, what might be needed are well designed comparative studies of online, in‐person, and blended interventions that are specified and that assess statistical equivalence rather than superiority with nested cost effectiveness assessment of the interventions themselves and healthcare utilisation downstream of the signature surgery.

### 4.3 Preliminary efficacy

The clinical improvement in physical function provides preliminary evidence of efficacy, although our results should be interpreted carefully in an implementation pilot using a small sample with no control group. Nonetheless, our results reflect the PEAK program's potential in keeping with its endorsement by the international osteoarthritis research society.^71^

We observed a significant and clinically meaningful (>5 points^72^) improvement in median OKS between t0 and t2 which is reassuring given that the OKS was designed to measure the effect of TKR on knee function and pain. While post‐hoc analysis showed there was no clinically significant improvement in OKS between t0 and t1 (median difference +4), OKS did not reduce either. Future randomised pilot work would determine whether prehabilitation maintains or improves OKS before surgery compared to receiving no prehabilitation and how this affects post‐surgery function.

We observed statistically significant increases in gait velocity including clinically meaningful (>0.1 m.s^‐1^ ^73^) improvement between t0 and t1. A previous randomised controlled trial reported similar gait velocities (1.3–1.4 m.s^‐1^) in its knee OA participants with comparable age, sex and BMI characteristics to ours.^74^ At post‐surgery (t2), we observed a reduction in gait velocity which is probably associated with adopting more cautious postsurgical gait,^75^ and the measurement point being relatively early (~3months) as iatrogenic reductions in gait speeds after surgery may take several months to improve.^76^

Significant improvement in anxiety was not replicated for depression. While the association between, and predisposition towards, anxiety, depression, and end‐stage OA pain is complex,^77^ both anxiety and depression have been observed to decrease postoperatively as a result of joint arthoplasty.^78^ So, it is unclear why we did not observe significant changes in depressive symptoms and could simply be a function of our small sample size. Smoking and depression have been found to be associated with worse TKR outcome (increased LOS).^79^ Our sample included one preoperative smoker. While we cannot conclude that the patient quit smoking due to our intervention, previous studies have observed higher quit rates if smoking cessation support is offered.^80^ There are compelling reports of COVID‐19 era delayed surgeries leading to increased opioid use. In contrast, only one participant in our study switched from no analgesia to a low opioid dose post‐surgery. Opioids have been a convenient solution in managing knee OA pain^81^ and with few other options available, prehabilitation that targets pain behaviours offers an alternative.

Participants’ BMI overall remained stable across time including two classified as obese. Our qualitative data revealed they did not choose to alter their behaviour despite the intervention. One perceived their diet was well‐balanced, while the other lacked effective strategies despite wanting to lose weight before surgery. This aligns with evidence that a comprehensive understanding of the distinct notions of individuals’ motivations are required if interventions are to result in effective weight loss.^82^

### 4.4 Strengths & weaknesses

Deploying laboratory‐standard and/or expert‐delivered interventions in the pilot‐to‐efficacy/effectiveness pipeline has been criticised^83^ because it affects external validity, risking generalisability at the efficacy stage.^84^ More real‐world pilots are the alternative.^85^ We adopted an ecologically valid COVID‐19 pandemic waiting list and utilised local infrastructure, equipment, and processes which is a strength of our study. But we deployed one expert clinician to deliver the intervention. This is a weakness because another clinician(s) could affect the acceptability of the intervention. Patients receiving knee revision surgeries were not included because we were unsure about the effect awaiting revisions has on comorbidities, and whether preoperative health expectations and behaviours may differ significantly. We therefore limited our inclusion to primary unilateral elective TKRs. However, it is possible that less strict inclusion criteria that included revisions would not have affected some of our implementation and fidelity assessments. We therefore acknowledge that including revisions would have improved our sample size for this pilot implementation study. The time between end of intervention (t1) and surgery date was uncontrolled. Therefore, t2‐t3 time was variable between participants. To offset these weaknesses, we propose to conduct another pilot study to assess the consistency of the intervention delivery and patients’ responses to it using a representative sample, and a control (waiting list) group to detect the effect size of the intervention and calculate a more accurate sample size for an experimental efficacy study which will preclude suitable effectiveness trials.^86^

## 5 CONCLUSION

This initial pilot study assumed that online multi‐modal prehabilitation could prevent patients awaiting TKR surgery from languishing at home. We have shown that a specified online intervention is acceptable, implementable, and shows evidence of preliminary efficacy. However, results show that the theory may hold for patients who were prioritised for surgery and who may not represent the spectrum of patients with frailty, obesity, poor QOL, socioeconomic status, and associated comorbidity, and further pilot work is indicated.

## CONFLICT OF INTEREST STATEMENT

The authors declare no conflicts of interest.

## Supporting information

Supporting information.

## Data Availability

The data that support the findings of this study are available on request from the corresponding author. The data are not publicly available due to privacy or ethical restrictions.

## References

[1] Collins NJ , Roos EM . Patient‐Reported outcomes for total hip and knee arthroplasty. Clin Geriatr Med. Aug 2012;28(3):367‐394. 10.1016/j.cger.2012.05.007 22840304

[2] National Joint Registry (NJR) Editorial Board. 20th annual report 2023: National Joint Registry for England, Wales, Northern Ireland and the Isle of Man. Accessed 17 August, 2020. https://reports.njrcentre.org.uk/

[3] National Joint Registry (NJR) Editorial Board. National Joint Registry for England, Wales, Northern Ireland and the Isle of Man 15th Annual Report. Pad Creative Ltd. for Health Quality Improvement Partnership (HQIP). Accessed 23 June, 2020. https://www.hqip.org.uk/resource/national-joint-registry-15th-annual-report-2018/

[4] Desmeules F , Dionne CE , Belzile ÉL , Bourbonnais R , Frémont P . The impacts of pre‐surgery wait for total knee replacement on pain, function and health‐related quality of life six months after surgery. J Eval Clin Pract. Feb 2012;18(1):111‐120. 10.1111/j.1365-2753.2010.01541.x 21040250

[5] Ragni E , Mangiavini L , Viganò M , et al. Management of osteoarthritis during the COVID‐19 pandemic. Clin Pharm Ther. Oct 2020;108(4):719‐729. 10.1002/cpt.1910 PMC7280639 32438454

[6] Song J , Chang RW , Dunlop DD . Population impact of arthritis on disability in older adults. Arthritis Care Res. Apr 15 2006;55(2):248‐255. 10.1002/art.21842 PMC2757646 16583415

[7] Xue QL . The frailty syndrome: definition and natural history. Clin Geriatr Med. Feb 2011;27(1):1‐15. 10.1016/j.cger.2010.08.009 21093718 PMC3028599

[8] Collard RM , Boter H , Schoevers RA , Oude Voshaar RC . Prevalence of frailty in community‐dwelling older persons: a systematic review. J Am Geriatr Soc. Aug 2012;60(8):1487‐1492. 10.1111/j.1532-5415.2012.04054.x 22881367

[9] Wang HT , Fafard J , Ahern S , Vendittoli PA , Hebert P . Frailty as a predictor of hospital length of stay after elective total joint replacements in elderly patients. BMC Musculoskelet Disord. Jan 16 2018;19(1):14. 10.1186/s12891-018-1935-8 29338705 PMC5771036

[10] Koponen MPH , Bell JS , Karttunen NM , Nykänen IA , Desplenter FAM , Hartikainen SA . Analgesic use and frailty among community‐dwelling older people: a population‐based study. Drugs Aging. Feb 2013;30(2):129‐136. 10.1007/s40266-012-0046-8 23288603

[11] Angst MS , Clark JD . Opioid‐induced hyperalgesia. Anesthesiology. Mar 2006;104(3):570‐587. 10.1097/00000542-200603000-00025 16508405

[12] Smith SR , Katz JN , Collins JE , et al. Cost‐Effectiveness of tramadol and oxycodone in the treatment of knee osteoarthritis. Arthritis Care Res. Feb 2017;69(2):234‐242. 10.1002/acr.22916 PMC5378156 27111538

[13] Public Helath England . COVID‐19: guidance on shielding and protecting people defined on medical grounds as extremely vulnerable. Government Digital Service. Updated 23 June 2020. Accessed 23 June, 2020. https://www.gov.uk/government/publications/guidance-on-shielding-and-protecting-extremely-vulnerable-persons-from-covid-19

[14] Oliveira MR , Sudati IP , Konzen VDM , et al. Covid‐19 and the impact on the physical activity level of elderly people: a systematic review. Exp Geront. Mar 2022;159:111675. 10.1016/j.exger.2021.111675 PMC8695515 34954282

[15] Smith TO , Dainty JR , MacGregor AJ . Changes in social isolation and loneliness following total hip and knee arthroplasty: longitudinal analysis of the English longitudinal study of ageing (ELSA) cohort. Osteoarthritis Cartilage. Sep 2017;25(9):1414‐1419. 10.1016/j.joca.2017.04.003 28445775

[16] Landeiro F , Leal J , Gray AM . The impact of social isolation on delayed hospital discharges of older hip fracture patients and associated costs. Osteoporos Int. Feb 2016;27(2):737‐745. 10.1007/s00198-015-3293-9 26337517

[17] Carli F , Zavorsky GS . Optimizing functional exercise capacity in the elderly surgical population. Curr Opin Clin Nutr Metab Care. Jan 2005;8(1):23‐32. 10.1097/00075197-200501000-00005 15585997

[18] Stowers MDJ , Manuopangai L , Hill AG , Gray JR , Coleman B , Munro JT . Enhanced recovery after surgery in elective hip and knee arthroplasty reduces length of hospital stay. ANZ J Surg. Jun 2016;86(6):475‐479. 10.1111/ans.13538 27018137

[19] Li C , Carli F , Lee L , et al. Impact of a trimodal prehabilitation program on functional recovery after colorectal cancer surgery: a pilot study. Surg Endosc. Apr 2013;27(4):1072‐1082. 10.1007/s00464-012-2560-5 23052535

[20] van Rooijen S , Carli F , Dalton S , et al. Multimodal prehabilitation in colorectal cancer patients to improve functional capacity and reduce postoperative complications: the first international randomized controlled trial for multimodal prehabilitation. BMC Cancer. Jan 22 2019;19(1):98. 10.1186/s12885-018-5232-6 30670009 PMC6341758

[21] Centre for Perioperative Care (CPOC) . Tackling the elective surgery backlog; Perioperative care solutions to the waiting list. CPOC. Accessed 26 February, 2024. https://www.cpoc.org.uk/sites/cpoc/files/documents/2021-09/CPOC-Perioperative-Care-Solutions-FINAL.pdf

[22] Zhu S , Qian W , Jiang C , Ye C , Chen X . Enhanced recovery after surgery for hip and knee arthroplasty: a systematic review and meta‐analysis. Postgrad Med J. Dec 2017;93(1106):736‐742. 10.1136/postgradmedj-2017-134991 28751437 PMC5740550

[23] National Institute for Health and Care Excellence (NICE) . Joint replacement (primary): hip, knee and shoulder [NICE guideline NG157]. Evidence review [C] for preoperative rehabilitation: Intervention evidence review underpinning recommendation 1.2.1 and the research recommendation in the NICE guideline. NICE. Updated June 2020. Accessed 18 April, 2021. https://www.nice.org.uk/guidance/ng157/chapter/Recommendations#preoperative-rehabilitation

[24] Whyte J , Hart T . It's more than a black box; it's a Russian doll: defining rehabilitation treatments. Am J Phys Med Rehabil. Aug 2003;82(8):639‐652. 10.1097/01.PHM.0000078200.61840.2D 12872021

[25] Hart T , Dijkers MP , Whyte J , et al. A Theory‐Driven system for the specification of rehabilitation treatments. Arch Phys Med Rehabil. Jan 2019;100(1):172‐180. 10.1016/j.apmr.2018.09.109 30267669

[26] Edwards R , Gibson J , Mungin‐Jenkins E , Pickford R , Lucas JD , Jones GD . A preoperative spinal education intervention for spinal fusion surgery designed using the rehabilitation treatment specification system is safe and could reduce hospital length of stay, normalize expectations, and reduce anxiety: a prospective cohort study. Bone Joint Open. Feb 2022;3(2):135‐144. 10.1302/2633-1462.32.BJO-2021-0160.R1 35139643 PMC8886324

[27] Seron P , Oliveros MJ , Gutierrez‐Arias R , et al. Effectiveness of telerehabilitation in physical therapy: A rapid overview. Phys Ther. Jun 1 2021;101(6):1‐18. 10.1093/ptj/pzab053 PMC7928601 33561280

[28] Suso‐Martí L , La Touche R , Herranz‐Gómez A , Angulo‐Díaz‐Parreño S , Paris‐Alemany A , Cuenca‐Martínez F . Effectiveness of telerehabilitation in physical therapist practice: an umbrella and mapping review with Meta‐Meta‐Analysis. Phys Ther. May 4 2021;101(5):1‐9. 10.1093/ptj/pzab075 PMC7928612 33611598

[29] Cottrell MA , Galea OA , O'Leary SP , Hill AJ , Russell TG . Real‐time telerehabilitation for the treatment of musculoskeletal conditions is effective and comparable to standard practice: a systematic review and meta‐analysis. Clin Rehabil. May 2017;31(5):625‐638. 10.1177/0269215516645148 27141087

[30] Ackerman IN , Buchbinder R , Osborne RH . Factors limiting participation in arthritis self‐management programmes: an exploration of barriers and patient preferences within a randomized controlled trial. Rheumatology. Mar 2013;52(3):472‐479. 10.1093/rheumatology/kes295 23148089

[31] March L , Amatya B , Osborne RH , Brand C . Developing a minimum standard of care for treating people with osteoarthritis of the hip and knee. Best Practice & Res Clin Rheumatol. Feb 2010;24(1):121‐145. 10.1016/j.berh.2009.10.002 20129205

[32] Grona SL , Bath B , Busch A , Rotter T , Trask C , Harrison E . Use of videoconferencing for physical therapy in people with musculoskeletal conditions: A systematic review. J Telemed Telecare. Jun 2018;24(5):341‐355. 10.1177/1357633X17700781 28403669

[33] Skivington K , Matthews L , Simpson SA , et al. A new framework for developing and evaluating complex interventions: update of medical research council guidance. BMJ. Sep 30 2021;374:n2061. 10.1136/bmj.n2061 34593508 PMC8482308

[34] Kellogg WK Foundation. Logic model development guide. https://wkkf.issuelab.org/resource/guiding-program-direction-with-logic-models.html

[35] Scott JM , Nilsen TS , Gupta D , Jones LW . Exercise therapy and cardiovascular toxicity in cancer. Circulation. Mar 13 2018;137(11):1176‐1191. 10.1161/CIRCULATIONAHA.117.024671 29530893 PMC6028047

[36] Pinnock H , Barwick M , Carpenter CR , et al. Standards for reporting implementation studies (StaRI): explanation and elaboration document. BMJ Open. Apr 3 2017;7(4):e013318. 10.1136/bmjopen-2016-013318 PMC5387970 28373250

[37] Pinnock H , Barwick M , Carpenter CR , et al. Standards for reporting implementation studies (StaRI) statement. BMJ. Mar 6 2017;356:i6795. 10.1136/bmj.i6795 28264797 PMC5421438

[38] Bellelli G , Morandi A , Davis DHJ , et al. Validation of the 4AT, a new instrument for rapid delirium screening: a study in 234 hospitalised older people. Age Ageing. Jul 2014;43(4):496‐502. 10.1093/ageing/afu021 24590568 PMC4066613

[39] National Institute for Health and Care Excellence (NICE) . Joint replacement (primary): hip, knee and shoulder (NICE guideline [NG157]). NICE. Updated 05 June 2020 Accessed 18 April, 2021. https://www.nice.org.uk/guidance/ng157 32881469

[40] Anderson AM , Comer C , Smith TO , et al. Consensus on pre‐operative total knee replacement education and prehabilitation recommendations: a UK‐based modified delphi study. BMC Musculoskelet Disord. Apr 14 2021;22(1):352. 10.1186/s12891-021-04160-5 33853564 PMC8044503

[41] Hinman R , Bennell K PEAK program ‐ on‐line training for physiotherapists in telehealth delivery of evidence‐based knee osteoarthritis care. The University of Melbourne. Updated 1 April 2020. Accessed 21 July, 2020. https://healthsciences.unimelb.edu.au/departments/physiotherapy/about-us/chesm/news-and-events/peak-training-program

[42] National Institute for Clinical Excellence (NICE) . Stop smoking interventions and services. NICE. Accessed 29 July, 2020. www.nice.org.uk/guidance/ng92

[43] Papadakis S , Cole AG , Reid RD , et al. Increasing rates of tobacco treatment delivery in primary care practice: evaluation of the Ottawa model for smoking cessation. Ann Fam Med. May 2016;14(3):235‐243. 10.1370/afm.1909 27184994 PMC4868562

[44] National Institute for Clinical Excellence (NICE) . Weight management: lifestyle services for overweight or obese adults. NICE. Accessed 29 July, 2020. https://www.nice.org.uk/guidance/ph53

[45] National Institute for Clinical Excellence (NICE) . Nutrition support for adults: oral nutrition support, enteral tube feeding and parenteral nutrition. NICE. Accessed 29 July, 2020. https://www.nice.org.uk/guidance/cg32 31999417

[46] Saunders RP , Evans MH , Joshi P . Developing a process‐evaluation plan for assessing health promotion program implementation: a how‐to guide. Health Promot Pract. Apr 2005;6(2):134‐147. 10.1177/1524839904273387 15855283

[47] Damschroder LJ , Aron DC , Keith RE , Kirsh SR , Alexander JA , Lowery JC . Fostering implementation of health services research findings into practice: a consolidated framework for advancing implementation science. Implement Sci. Aug 7 2009;4:50. 10.1186/1748-5908-4-50 19664226 PMC2736161

[48] Sanetti LM , Cook BG , Cook L . Treatment fidelity: what it is and why it matters. Learn Disabil Res Pract. 2021;36(1):5‐11.

[49] Murray DW , Fitzpatrick R , Rogers K , et al. The use of the Oxford hip and knee scores. J Bone Joint Surg Br. Aug 2007;89(8):1010‐1014. 10.1302/0301-620X.89B8.19424 17785736

[50] Fess EE . Grip strength. In: Casanova JS , ed. Clinical assessment recommendations. 2nd ed. American Society of Hand Therapists; 1992:41‐45.

[51] Bohannon RW , Peolsson A , Massy‐Westropp N , Desrosiers J , Bear‐Lehman J . Reference values for adult grip strength measured with a jamar dynamometer: a descriptive meta‐analysis. Physiotherapy. 2006;92(1):11‐15. 10.1016/j.physio.2005.05.003

[52] Strassmann A , Steurer‐Stey C , Lana KD , et al. Population‐based reference values for the 1‐min sit‐to‐stand test. Int J Public Health. Dec 2013;58(6):949‐953. 10.1007/s00038-013-0504-z 23974352

[53] Cohen H , Blatchly CA , Gombash LL . A study of the clinical test of sensory interaction and balance. Phys Ther. Jun 1993;73(6):346‐351. discussion 351‐4 10.1093/ptj/73.6.346 8497509

[54] Perera S , Patel KV , Rosano C , et al. Gait speed predicts incident disability: A pooled analysis. J Gerontol A Biol Sci Med Sci. 2016;71(1):63‐71. 10.1093/gerona/glv126 26297942 PMC4715231

[55] Snaith RP , Zigmond AS . The hospital anxiety and depression scale. BMJ. Feb 1 1986;292(6516):344. 10.1136/bmj.292.6516.344 PMC1339318 3080166

[56] Stern AF . The hospital anxiety and depression scale. Occup Med. Jul 2014;64(5):393‐394. 10.1093/occmed/kqu024 25005549

[57] EuroQol Research Foundation . EQ‐5D‐5L User Guide. EuroQol Research Foundation. Accessed 29 July, 2020. https://euroqol.org/publications/user-guides

[58] Kidner CL , Mayer TG , Gatchel RJ . Higher opioid doses predict poorer functional outcome in patients with chronic disabling occupational musculoskeletal disorders. J Bone Joint Surg Am Volume. Apr 2009;91(4):919‐927. 10.2106/JBJS.H.00286 PMC2665041 19339577

[59] Friedman M . The use of ranks to avoid the assumption of normality implicit in the analysis of variance. J Am Stat Assoc. Dec 1937;32(200):675‐701. 10.2307/2279372

[60] Cochran WG . The comparison of percentages in matched samples. Biometrika. Dec 1950;37(3‐4):256‐266. 14801052

[61] Bree RT , Gallagher G . Using microsoft excel to code and thematically analyse qualitative data: a simple, cost‐effective approach. Ireland J Higher Educ. 2016;8(2):2811‐2824.

[62] Plummer P . Focus group methodology. part 2: considerations for analysis. Int J Ther Rehabil. 2017;24(8):345‐351.

[63] National Joint Registry . Knees ‐ Primary Procedures ‐ Patient Characteristics for Primary Knee Replacement Procedures. Accessed 18 February, 2024. https://reports.njrcentre.org.uk/knees-primary-procedures-patient-characteristics

[64] National Joint Registry (NJR) Editorial Board. National Joint Registry for England, Wales, Northern Ireland and the Isle of Man 18th Annual Report. Pad Creative Ltd. for Health Quality Improvement Partnership (HQIP). Accessed 14 August, 2023. https://www.hqip.org.uk/resource/njr-18th-annual-report-2021/

[65] Boer P . Estimated lean body mass as an index for normalization of body fluid volumes in humans. Am J Physiol Renal Physiol. Oct 1984;247(4 Pt 2):F632‐F636. 10.1152/ajprenal.1984.247.4.F632 6496691

[66] Charlson ME , Pompei P , Ales KL , MacKenzie CR . A new method of classifying prognostic comorbidity in longitudinal studies: development and validation. J Chronic Dis. 1987;40(5):373‐383. 10.1016/0021-9681(87)90171-8 3558716

[67] Perepletchikova F , Kazdin AE . Treatment integrity and therapeutic change: issues and research recommendations. Clin Psychol Sci Pract. 2005;12(4):365‐383.

[68] Runhaar J , Holden MA , Hattle M , et al. Mechanisms of action of therapeutic exercise for knee and hip OA remain a black box phenomenon: an individual patient data mediation study with the OA trial bank. RMD Open. Aug 2023;9(3):e003220. 10.1136/rmdopen-2023-003220 37640513 PMC10462947

[69] Latulippe K , Hamel C , Giroux D . Social health inequalities and ehealth: A literature review with qualitative synthesis of theoretical and empirical studies. J Med Internet Res. Apr 27 2017;19(4):e136. 10.2196/jmir.6731 28450271 PMC5427250

[70] Tsang MP , Man GCW , Xin H , Chong YC , Ong MTY , Yung PSH . The effectiveness of telerehabilitation in patients after total knee replacement: A systematic review and meta‐analysis of randomized controlled trials. J Telemed Telecare. Jun 2024;30(5):795‐808. 10.1177/1357633X221097469 35549756

[71] Quicke JG , Swaithes LR , Campbell LH , et al. The OARSI “joint effort initiative” repository of online osteoarthritis management programmes: an implementation rapid response during covid‐19. Osteoarthritis Cartilage. 2021/04/01/2021;29:S87‐S89. 10.1016/j.joca.2021.02.121

[72] Clement ND , MacDonald D , Simpson AHRW . The minimal clinically important difference in the Oxford knee score and short form 12 score after total knee arthroplasty. Knee Surg Sports Traumatol Arthrosc. 2014/08/01 2014;22(8):1933‐1939. 10.1007/s00167-013-2776-5 24253376

[73] Perera S , Mody SH , Woodman RC , Studenski SA . Meaningful change and responsiveness in common physical performance measures in older adults. J Am Geriatr Soc. May 2006;54(5):743‐749. 10.1111/j.1532-5415.2006.00701.x 16696738

[74] Henriksen M , Klokker L , Bartholdy C , Schjoedt‐Jorgensen T , Bandak E , Bliddal H . No effects of functional exercise therapy on walking biomechanics in patients with knee osteoarthritis: exploratory outcome analyses from a randomised trial. BMJ Open Sport Exerc Med. 2017;2(1):bmjsem‐2017‐000230. 10.1136/bmjsem-2017-000230 PMC5569270 28879038

[75] Fallah Yakhdani HR , Bafghi HA , Meijer OG , et al. Stability and variability of knee kinematics during gait in knee osteoarthritis before and after replacement surgery. Clin Biomech. Mar 2010;25(3):230‐236. 10.1016/j.clinbiomech.2009.12.003 20060628

[76] Abbasi‐Bafghi H , Fallah‐Yakhdani HR , Meijer OG , et al. The effects of knee arthroplasty on walking speed: a meta‐analysis. BMC Musculoskelet Disord. May 6 2012;13:66. 10.1186/1471-2474-13-66 22559793 PMC3481434

[77] Jiménez Ortiz M , Espinosa Ruiz A , Martínez Delgado C , Barrena Sánchez P , Salido Valle JA . Do preoperative anxiety and depression influence the outcome of knee arthroplasty? Reumatol Clin (Engl Ed). May‐Jun 2020;16(3):216‐221. ¿Influye la ansiedad y depresión preoperatorias en los resultados de la artroplastia de rodilla? 10.1016/j.reuma.2018.06.008 30057293

[78] Duivenvoorden T , Vissers MM , Verhaar JAN , et al. Anxiety and depressive symptoms before and after total hip and knee arthroplasty: a prospective multicentre study. Osteoarthritis Cartilage. 2013/12/01/2013;21(12):1834‐1840. 10.1016/j.joca.2013.08.022 24012622

[79] Halawi MJ , Chiu D , Gronbeck C , Savoy L , Williams VJ , Cote MP . Psychological distress independently predicts prolonged hospitalization after primary total hip and knee arthroplasty. J Arthroplasty. Aug 2019;34(8):1598‐1601. 10.1016/j.arth.2019.03.063 31005432

[80] Cropley M , Theadom A , Pravettoni G , Webb G . The effectiveness of smoking cessation interventions prior to surgery: a systematic review. Nicotine Tob Res. Mar 2008;10(3):407‐412. 10.1080/14622200801888996 18324557

[81] Farrow L , Gardner WT , Tang CC , Low R , Forget P , Ashcroft GP . Impact of COVID‐19 on opioid use in those awaiting hip and knee arthroplasty: a retrospective cohort study. BMJ Qual Safety. Aug 2023;32(8):479‐484. 10.1136/bmjqs-2021-013450 34521769

[82] Poraj‐Weder M , Wąsowicz G , Pasternak A . Why it is so hard to lose weight? an exploration of patients’ and dietitians’ perspectives by means of thematic analysis. Health Psychol Open. Jan‐Jun 2021;8(1):20551029211024406. 10.1177/20551029211024406 34211722 PMC8216368

[83] Beets MW , Weaver RG , Ioannidis JPA , et al. Identification and evaluation of risk of generalizability biases in pilot versus efficacy/effectiveness trials: a systematic review and meta‐analysis. Int J Behav Nutrition Phys Activity. 2020/02/11 2020;17(1):19. 10.1186/s12966-020-0918-y PMC7014944 32046735

[84] Green LW , Glasgow RE . Evaluating the relevance, generalization, and applicability of research: issues in external validation and translation methodology. Eval Health Prof. Mar 2006;29(1):126‐153. 10.1177/0163278705284445 16510882

[85] World Health Organization (WHO) ExpandNet . Begining with the End in Mind: Planning pilot projects and other programmatic research for sucessful scaling up. WHO. Accessed 20 February, 2024. https://apps.who.int/iris/bitstream/handle/10665/44708/9789241502320_eng.pdf;jsessionid=F51B37DE2EF6215F95067CD7C13D4234?sequence=1.

[86] West MA , Jack S , Grocott MPW . Prehabilitation before surgery: is it for all patients? Best Pract Res Clin Anaesthesiol. Dec 2021;35(4):507‐516. 10.1016/j.bpa.2021.01.001 34801213

