# Supplementary material for: Introducing a specified on‐line multimodal prehabilitation approach for total knee replacement surgery candidates using data from the COVID‐19 pandemic: An exploratory field‐based, pre‐post, mixed methods implementation pilot study
Source: J Eval Clin Pract. 2024 Oct 16;31(3):e14186. doi: 10.1111/jep.14186 (PMC12021319; doi:10.1111/jep.14186)
Supplement: Supplementary file 1 — Supporting information. [file JEP-31-0-s001.docx]

# Supplementary Material

## Specified Interventions using the RTSS^1^

### Cardiovascular exercise and strength & balance training

The program targets increasing knee muscle strength, balance, and reducing pain with the downstream aim of improving gait speed and QOL. Exercise programmes were prescribed based upon patient’s baseline measures, their self-rated exertion (scale 0-10, 0=rest, 10=maximal exertion). Training and education booklets were provided as ingredients, alongside use of the PEAK program which employs ingredients to enhance volitional performance of unsupervised exercise. Patients had a telephone follow up at week 2, 4, 6 and 8 where their exercises were reviewed, progressed and further exercises were added at week 2, 4 and 6. Patients were encouraged to continue their exercise programmes up until their surgery

Supplementary Table 1: PEAK program specification based on the RTSS^1^
*Target groups – O (Organ functions), S&H (Skills and Habits), or R (Representations). Organ functions are treatments in which the functions of organs or organ systems are modified, often by systematic stimulation, Skills and Habits are treatments that have in common learning or improving a skill via practicing, or reducing the effort required/increasing the habitual nature of a behavioural routine, Representations are treatments aimed at changing internal (i.e., central nervous system) representations related to cognitions, affect, motivation, and intentions to perform volitional behaviours.
†Volition Type - a description of whether the treatment target is not dependent on volitional effort (NV – non-volitional) or whether it is dependent on volitional effort on the part of the patient (or other specified person e.g. carer), and if so whether the therapists directs volitional behaviours by being present (DV – direct volitional) or not when the patient is responsible to perform or deploy the treatment volitionally independent of the therapist (V – volitional); #Mechanism of Action – the process by which a treatment’s active ingredients induce change in the target of treatment;**Ingredients – the observable, measurable and active elements of treatment that are hypothesized to directly change the target of treatment; ||Exercise dosing was recommended using internationally recognised guidelines;^2^ OA - Osteoarthritis

| Target | | | #Mechanism of Action | Ingredient | |
| --- | --- | --- | --- | --- | --- |
| What/in what way | *Group | †Volition |  | **Ingredients | \|\|Dosing Parameters |
| Lower limb strength/Increase | O | DV | Muscle fibre hypertrophy | Examples of home-based strength exercises tailored to the capabilities and opportunities available to patients, including:  Sit to stands from chair  Supine bridging on bed  Hip abduction in side lying  Knee extension in sitting  Inner range quads in long sitting  Lunges with chair support  Step ups  Wall squats | 8 weeks  3x weekly  3-4 sets (tailored to patient)  6-15 reps (tailored to patient)  Progressions including (tailored to patient)  Removal of use of hands for Sit to stand  Addition of ankle weight for open chain exercises  Removal of chair support for lunges  Addition of up to 2 exercises, with max 6 overall |
| Static balance of symptomatic leg / Increase | S&H | DV | Learning by doing | Example of home-based balance exercises tailored to the capabilities and opportunities available to patients, including:  Single leg balance eyes open on symptomatic leg | 8 weeks  3x weekly  3-4 sets (tailored to patient)  2-5 reps (tailored to patient)  30 second holds (tailored to patient)  Progressions including (tailored to patient)  Closed eyes |
| Performance of home exercise program / As directed | R | V | Cognitive information processing | Tailoring of exercises to capabilities and opportunities of patient  Therapist prompted discussion of patient experiences of exercises  Congratulate adherence  Discuss reasons for non-adherence and troubleshooting  Discussion of benefit towards management of OA  Watched performance of patient performing exercise to ensure working at correct intensity and correct technique  Check patient knows how to alter/progress exercises beyond program  Verbal encouragement to continue programme in future | Performed at weeks, 1, 2, 4, 7 and 10 |
| Propensity to perform physical activity / increase | R | V | Cognitive information processing | Use of activity tracker if patient in possession  Use of logbook for recording daily steps/type and amount of physical activity  Setting of daily step goal  Therapist and patient agreed activity plan to achieve goal  Discussion of benefit towards management of OA  Ask patient to identify potential barriers and supported planning of strategies to overcome them  Check patient knows how to alter/progress exercises beyond program  Verbal encouragement to continue program in future | Performed at weeks, 1, 2, 4, 7 and 10 |

### Smoking cessation

Supplementary Table 2: Smoking cessation specification based on the RTSS^1^

| Target | | | #Mechanism of Action | Ingredient | |
| --- | --- | --- | --- | --- | --- |
| What/in what way | *Group | †Volition |  | **Ingredients | Dosing parameters |
| Propensity to quit smoking / Increase | R | V | Cognitive information processing | Provision of local document leaflet “stop before your op”‡ detailing benefits of giving up  Asking if the patient smokes  Verbal education about the risks associated with smoking  Act upon patient response to local smoking cessation service  Patients not currently smoking, rewarded with therapist praise | Performed at weeks, 1, 2, 4, 7 and 10 |

*Target groups –R (Representations). Representations are treatments aimed at changing internal (i.e., central nervous system) representations related to cognitions, affect, motivation, and intentions to perform volitional behaviours; †Volition Type - a description of whether the treatment target is not dependent on volitional effort (NV – non-volitional) or whether it is dependent on volitional effort on the part of the patient (or other specified person e.g. carer), and if so whether the therapists directs volitional behaviours by being present (DV – direct volitional) or not when the patient is responsible to perform or deploy the treatment volitionally independent of the therapist (V – volitional); #Mechanism of Action – the process by which a treatment’s active ingredients induce change in the target of treatment. **Ingredients – the observable, measurable and active elements of treatment that are hypothesised to directly change the target of treatment; ‡the ‘Stop before your op’ Guy’s and St Thomas’ patient leaflet which includes information about why it is important to give up, benefits of giving up, and expected timescales of benefits

### Opioid use and pain management

Supplementary Table 3: Opioid use modulation specification based on the RTSS^1^

*Target groups –R (Representations). Representations are treatments aimed at changing internal (i.e., central nervous system) representations related to cognitions, affect, motivation, and intentions to perform volitional behaviours;†Volition Type - a description of whether the treatment target is not dependent on volitional effort (NV – non-volitional) or whether it is dependent on volitional effort on the part of the patient (or other specified person e.g. carer), and if so whether the therapists directs volitional behaviours by being present (DV – direct volitional) or not when the patient is responsible to perform or deploy the treatment volitionally independent of the therapist (V – volitional); #Mechanism of Action – the process by which a treatment’s active ingredients induce change in the target of treatment. **Ingredients – the observable, measurable and active elements of treatment that are hypothesised to directly change the target of treatment; §as per RTSS guidance, the table includes a dosing column - note however that for all treatment components, dosing was notated as not applicable - this is due to there being no quantifiable significance in the delivery of ingredients that could be hypothesised to affect the outcome of the specified treatment targets; GP – General Practitioner, OA – Osteoarthritis.

| Target | | | #Mechanism of Action | Ingredient | |
| --- | --- | --- | --- | --- | --- |
| What/in what way | *Group | †Volition |  | **Ingredients | §Dosing parameters |
| Knowledge of risks associated with high use of opioids/Increase | R | DV | Cognitive information processing | Verbal education about the detrimental effect and risks associated with high use.  Written information leaflet  Verbal encouragement to seek GP input if high usage and had not already done so in last 3 months | Not applicable |
| Knowledge of OA pain management/Increase | R | DV | Cognitive information processing | Therapist led verbal education about managing OA pain with suggested tailored strategies  Written Information leaflet about managing OA pain | Not applicable |

### Nutritional intake

Supplementary Table 4: Nutritional intake behaviour change specification based on the RTSS^1^

*Target groups –R (Representations). Representations are treatments aimed at changing internal (i.e., central nervous system) representations related to cognitions, affect, motivation, and intentions to perform volitional behaviours; †Volition Type - a description of whether the treatment target is not dependent on volitional effort (NV – non-volitional) or whether it is dependent on volitional effort on the part of the patient (or other specified person e.g. carer), and if so whether the therapists directs volitional behaviours by being present (DV – direct volitional) or not when the patient is responsible to perform or deploy the treatment volitionally independent of the therapist (V – volitional); #Mechanism of Action – the process by which a treatment’s active ingredients induce change in the target of treatment. **Ingredients – the observable, measurable and active elements of treatment that are hypothesised to directly change the target of treatment; §as per RTSS guidance, the table includes a dosing column - note however that for all treatment components, dosing was notated as not applicable - this is due to there being no quantifiable significance in the delivery of ingredients that could be hypothesised to affect the outcome of the specified treatment targets; OA – Osteoarthritis.

| Target | | | #Mechanism of Action | Ingredient | |
| --- | --- | --- | --- | --- | --- |
| What/in what way | *Group | †Volition |  | **Ingredients | §Dosing parameters |
| Knowledge of adult malnutrition management/Increase | R | DV | Cognitive information processing | Written information leaflets “A guide to managing malnutrition in the community” | Not applicable |
| Knowledge of relationship of weight loss and OA/Increase | R | DV | Cognitive information processing | Therapist led verbal education about effect of weight loss on OA  Written Information leaflet about effect of weight loss on OA | Not applicable |

## Contextual Factors

Supplementary Table 5: Local context summary based on CFIR^3^

| CFIR Topic/Description | | Summary |
| --- | --- | --- |
| I Intervention Characteristics | 1. Intervention Source 2. Evidence Strength & Quality 3. Relative advantage 4. Adaptability 5. Trialability 6. Complexity 7. Design Quality and Packaging 8. Cost | - The research team harbour attitudes toward and high value placed on multi-modal prehabilitation and are familiar with prehabilitative principles in their clinical roles - Peri-surgical multi-disciplinary team perception of the quality and validity of evidence for prehabilitation is high, although it is acknowledged less so for multi-modal prehabilitation because the mechanisms are less understood - There is no real comparative intervention other than no intervening with patients, therefore there is a high relative advantage perception among stakeholders while acknowledging the extra costs of the intervention and whether an online mode will be truly equitable - The intervention can be adapted, tailored, refined, or reinvented to meet local needs. - It is possible to test the intervention in the organization and reverse it if appropriate - The perceived difficulty of implementation is medium - The PEAK aspect of the intervention is of high quality in how it is bundled and presented, the other elements are to be determined - N/A - Costs (material, cognitive, or organizational) have not been considered in this project at this stage |
| II Outer Setting | 1. Patient Needs & Resources 2. Cosmopolitanism 3. Peer Pressure 4. External Policy & Incentives | - We assume there is appetite among all patients, representative of the diversity of local population, waiting for TKR to participate in prehabilitative interventions, there was no local lived experience data prior to the project - N/A - N/A - There is pressure in the organization to maximize tariff income on planned surgical interventions including reducing hospital length of stay and re-admission rates from signature surgical procedures |
| III Inner Setting | 1. Structural Characteristics 2. Networks & Communications 3. Culture 4. Implementation Climate 5. Tension for Change 6. Compatibility 7. Relative Priority 8. Organizational Incentives & Rewards 9. Goals and Feedback 10. Learning Climate 11. Readiness for Implementation 12. Leadership 13. Engagement 14. Available Resources | - The organization provides secondary and tertiary care serving a local population of 625,300.^4^ It is fully committed to improving the experiences of all staff and patients, by providing inclusive healthcare to patients, pledging to be an inclusive workplace and promise to workplace equality. The organization’s strategic choices paper has prioritized equality, diversity and inclusive approaches to offer all staff the opportunity for involvement in research design and delivery, and to meet the highest standards of research integrity. The local physiotherapy department has deployed a strategy since 2020 of its staff being research active including quality improvement, primary research, and implementation projects. - The quality of networks in the organization is high, its clinical group structure means there are risks that inter-group networks may not be as strong - The organization’s culture is to support implementing the highest evidence-based interventions for the patients it is responsible for - Stakeholders do not perceive an imminent need for change but welcome innovative attempts - The intervention fits with existing workflows and systems and is compatible with an increase in on-line interventions offered since the COVID pandemic - The shared perception from stakeholders is that the organization values implementation that reduces costs or workflows, but does not necessarily value implementation that has not been couched in these terms - N/A - N/A - N/A - N/A - N/A - N/A - N/A - N/A |
| IV Characteristics of Individuals | 1. Knowledge & Beliefs about the Intervention 2. Self-efficacy 3. Individual Stage of Change 4. Individual Identification with Organization 5. Other Personal Attributes | - High - High - The research team value the intervention and are ready to move from technical competency in delivering the intervention to mastery - The research team perceive the local higher management support to be committed to the implementation of the intervention - The research team host strong tolerance of ambiguity and can intellectually reason its position |
| V Process | 1. Planning 2. Engaging 3. Opinion Leaders 4. Formally appointed internal implementation leaders 5. Champions 6. External Change Agents 7. Executing 8. Reflecting & Evaluating | - The research team was able to plan to trial the use of the PEAK intervention in advance of the implementation project - No specific strategy utilizing social marketing, education, role modelling, training, or other similar activities was undertaken to attract other stakeholders during the duration of the project other than buy-in from surgical colleagues - The research team includes clinicians of sufficient seniority, credibility, and reputation who are thus able to influence attitudes and beliefs of colleagues towards implementation - None - Not appropriated for this project - Not appropriated for this project - The implementation project was carried out as planned within the resources committed to this preliminary work - No quantitative or qualitative feedback about the progress and quality of implementation accompanied with regular personal and team debriefing about progress and experience was planned during this project |

## Interview Topic Guide

Supplementary Table 6: Focus group topic guide
One participant who chose not to participate in the focus group reviewed the and suggested edits in language to make the group more relevant. The session started with an ice breaker to relax the participants and went on to open introductory broad questions about their experience of the intervention. Gradually the questions become more focused relating to the 4 different parts of the multimodal intervention and finished with asking if there was anything else that participants wished to add to the discussed topics or any other feedback that they wanted to provide.

| Can you please tell me about your experience of the physiotherapy rehab before your Total knee replacement surgery? |
| --- |
| Thinking about the information you received about knee arthritis / dietary advice, tell me what your thoughts on this were? |
| Thinking about the exercises completed during the program, tell me what you thought about them? |
| If you are a smoker, tell me if it changed your smoking habits? |
| Thinking about the delivery of the program being online, what did you think about that? Would you have preferred face to face classes? |
| Thinking about your experience of the rehabilitation program prior to surgery where there any specific issues, concerns, or problems that you faced during the program? |
| Is there anything else you want to add to the conversation about this program? |

# Supplementary Material References

1. Hart T, Dijkers MP, Whyte J, et al. A Theory-Driven System for the Specification of Rehabilitation Treatments. *Arch Phys Med Rehabil*. Jan 2019;100(1):172-180. doi:https://doi.org/10.1016/j.apmr.2018.09.109

2. Haskell WL, Lee I-M, Pate RR, et al. Physical activity and public health: updated recommendation for adults from the American College of Sports Medicine and the American Heart Association. *Circulation*. 2007;116(9):1081.

3. Damschroder LJ, Aron DC, Keith RE, Kirsh SR, Alexander JA, Lowery JC. Fostering implementation of health services research findings into practice: a consolidated framework for advancing implementation science. *Implement Sci*. Aug 7 2009;4:50. doi:10.1186/1748-5908-4-50

4. Office for National Statistics. How the population changed in Lambeth and Southwark: Census 2021. ONS. Accessed 30 May, 2023. https://www.ons.gov.uk/visualisations/censuspopulationchange/E09000028/
